# Can rural health insurance improve equity in health care utilization? a comparison between China and Vietnam

**DOI:** 10.1186/1475-9276-11-10

**Published:** 2012-02-29

**Authors:** Xiaoyun Liu, Shenglan Tang, Baorong Yu, Nguyen Khanh Phuong, Fei Yan, Duong Duc Thien, Rachel Tolhurst

**Affiliations:** 1 China Center for Health Development Studies, Peking University, PO box 505, 38 Xue Yuan Road, Hai Dian District, Beijing 100191, P. R. China; 2 Liverpool School of Tropical Medicine, Pembroke Place, Liverpool L3 5QA, UK; 3 Special Programme for Research and Training in Tropical Diseases (TDR), World Health Organization, Avenue Appia 20, 1211, Geneva 27, Switzerland; 4 Shandong University, 44 Wenhua Xi Road, Jinan 250012, Shandong, China; 5 Health Strategy and Policy Institute, 138 Giang Vo, Ha Noi, Viet Nam; 6 School of Public Health, Fudan University, 138 Yi Xue Yuan Road, Shanghai, China; 7 Health Policy Unit, Ministry of Health, 138A Giang Vo, Hanoi, Vietnam

**Keywords:** Health insurance, Rural area, Equity, Health services utilization, China, Vietnam

## Abstract

**Introduction:**

Health care financing reforms in both China and Vietnam have resulted in greater financial difficulties in accessing health care, especially for the rural poor. Both countries have been developing rural health insurance for decades. This study aims to evaluate and compare equity in access to health care in rural health insurance system in the two countries.

**Methods:**

Household survey and qualitative study were conducted in 6 counties in China and 4 districts in Vietnam. Health insurance policy and its impact on utilization of outpatient and inpatient service were analyzed and compared to measure equity in access to health care.

**Results:**

In China, Health insurance membership had no significant impact on outpatient service utilization, while was associated with higher utilization of inpatient services, especially for the higher income group. Health insurance members in Vietnam had higher utilization rates of both outpatient and inpatient services than the non-members, with higher use among the lower than higher income groups. Qualitative results show that bureaucratic obstacles, low reimbursement rates, and poor service quality were the main barriers for members to use health insurance.

**Conclusions:**

China has achieved high population coverage rate over a short time period, starting with a limited benefit package. However, poor people have less benefit from NCMS in terms of health service utilization. Compared to China, Vietnam health insurance system is doing better in equity in health service utilization within the health insurance members. However with low population coverage, a large proportion of population cannot enjoy the health insurance benefit. Mutual learning would help China and Vietnam address these challenges, and improve their policy design to promote equitable and sustainable health insurance.

## Introduction

Many developing countries are trying to find ways to achieve universal healthcare coverage, and reduce the reliance on out-of-pocket payment and provide financial protection against high medical expenses [1,2]. Tax-based health financing and social health insurance are most frequently used mechanisms for achieving the goal. Both China and Vietnam have experienced rapid economic development and dramatic social changes over the past three decades. Health sector reforms in the two countries have led health facilities to rely increasingly on user charges. This has resulted in great financial difficulties in accessing health care, especially for the rural poor [3,4]. The central governments of both countries have promoted the development of social health insurance for many years to address these problems. However, different historical and political trajectories have led to the development of very different rural health insurance policies and systems.

### Vietnamese health insurance system

Vietnam's national health insurance system is divided into compulsory health insurance (CHI) and voluntary health insurance (VHI). Both CHI and VHI are designed and managed by the central government, and have been experiencing rapid policy changes in the recent years [5]. The CHI system was initiated in 1992 and now covers mainly employees in the formal sector, civil servants, and some social protection groups. Since 2005, the poor and ethnic minorities have also been covered by the CHI. The eligibility of the poor for CHI is authorized by local government. The premium for the CHI is 3% of employees' salary, of which 2% is paid by employers, and 1% by employees. CHI premiums for the poor are paid by central government budget, and have increased from 50,000 VND (about 2.8 USD) per capita in 2005 to 180,000 VND (10 USD) in 2008. CHI has a clearly defined benefit package. Patients needed to co-pay 20% of their medical cost before 2005. This co-payment was cancelled after 2005 with the exception of high technology health services, for which CHI only covered a limited part of the cost. A new health insurance law (being effective on 1^st ^January 2010) again regulated a co-payment level at 5-20%.

VHI in Vietnam was originally designed to cover specific occupational and age groups such as school children, farmers, professional groups. A minimum enrollment rate of 10% was set for each group. In 2007, VHI scheme was made available to all citizens and the 10% minimum rate of enrollment was canceled. Premiums for VHI varied across groups and urban/rural residence: 25,000 to 70,000 VND (1.4 to 3.9 US dollars) for school children and 60,000 to 140,000 VND (3.3 to 7.8 US dollars) for other residents. Since 2007, VHI premium has increased sharply (from 60,000 to 120,000 VND for school children, and from 120,000 VND to 320,000 VND for all others). Government did not have subsidy for VHI. VHI had a same benefit package as CHI, but co-payment for high technology health services was 40%.

The Vietnam Social Security agency (VSS), established in 2003, is the government agency responsible for the administration of social insurance programmes including the CHI and VHI. VSS is responsible for collecting premium, pooling the fund, issuing health insurance cards and reimbursing service providers [5].

### Chinese health insurance system

China has developed separate health insurance systems for urban and rural areas. Urban residents are covered by an employment-based basic medical insurance scheme and an urban resident scheme [6]. In rural areas, the central government of China launched the New Medical Cooperative Scheme (NCMS) in 2003. This is a voluntary health insurance scheme. All NCMS members pay a flat rate premium of 10 Yuan (1.25 US dollar), which has increased to 20 Yuan since 2008. Central and local governments heavily subsidize NCMS to varying degrees in different regions and provinces. NCMS benefit package focuses on inpatient services, aiming to reduce financial burden due to high medical cost [7]. The responsibility of collecting and managing the NCMS fund are undertaken at county level. This, together with local governments' varying financial and management capacity, has resulted in great variations in local NCMS funding envelopes and policy designs [8].

Fee-For-Service was the most common provider payment method in both countries. Along with the health insurance programmes, both countries piloted other methods of provider payment such as capitation and diagnosis related group (DRG).

### Health insurance and equity in healthcare

Equity in healthcare, as one important component of the broad concept of health equity, is a multidimensional concept, which includes equal access to available care for equal need, and equal quality of care for all [9,10]. Many countries have been developing social health insurance as a main health financing mechanism to secure access to adequate health care for all at an affordable price [1]. However, there is evidence that the specific design of health insurance schemes within particular contexts influences their impact on equity in access to and utilization of health services [11-13]. One of the major concerns from an equity perspective is whether disadvantaged groups can benefit from health insurance in terms of improved access to services [14].

Using data collected as part of a research project aiming to contribute towards the development of equitable and sustainable rural health insurance in China and Vietnam (RHINCAV), this paper will focus on the access aspect of the "equity" concept. Access to healthcare is a complex concept, encompassing a number of different dimensions [9]. Two important indicators of access when analysing the impact of health insurance are utilisation of services and financial protection (which can be seen as a measure of affordability). In this paper we focus on utilisation as the major measure of access but we also explore some of the difficulties experienced by health insurance members in utilising services, including the degree of financial protection. The impact of rural health insurance on other aspects of health services will be addressed in other papers. This paper first presents health insurance coverage in the two countries and compare perceived health needs between health insurance members and non-members. Health care utilization between members and non-members in different income groups is compared. Experiences and views of the health insurance schemes by members and other relevant stakeholders are then presented to offer explanations and add depth to the quantitative findings. Finally, the implications for future policy and scheme development are discussed, with a consideration of what the two countries may learn from each other and the potential resonance of their experiences for other countries in economic transition.

## Methods

### Study design and sampling

China and Vietnam were chosen as study countries because they have similar social economic background and are facing similar challenges in financing their health system. Interestingly, they have however chosen different health insurance systems which may have different implications on equity in health care.

Two provinces from each country (Shandong and Ningxia from China, Hai Duong and Bac Giang from Vietnam) were selected for this study. The selection of provinces was based on three criteria: 1) one province represented relatively developed areas within the country (Shangdong and Hai Duong), and the other one represented less developed areas (Ningxia and Bac Giang); 2) all study sites had established a rural health insurance system; 3) local governments were capable and willing to cooperate with the study. In each province, 2 districts from Vietnam and 3 counties from China were selected for the study using similar selection criteria as those of provinces.

The study used a survey and qualitative methods to collect and analyze data. Data from the household survey was used to analyze and compare the utilization of health services between health insurance members and non-members in different income groups. This was then triangulated and explained through focus group discussions (FGD) with health insurance members and in-depth interviews with health insurance managers, local government leaders and administrators.

Sample size for household survey was calculated based on estimation and comparison of utilization rate of inpatient services between different economic groups. In China, a sample size of 22,008 individuals (11,004 per province) was estimated based on inpatient utilization rate in low income group (3.3%) and high income group (4.2%) from 2003 National Health Service Survey. In Vietnam, a smaller sample size of 7518 individuals was estimated based on an expected inpatient utilization rate of 5.2%, also with its smaller population size in consideration.

Multistage sampling processes were used in household survey. In China, we selected 3 townships from each county, 3 villages from each township, and then a systematic random sample of 100 households in each village were selected based on the household registration. Similar processes were used in Vietnam: 4 communes from each district, 3 villages from each commune, and a systematic random sample of 50 households from each village were selected from a village household registration list. In total, 6,147 households (22,636 individuals) in China and 2,397 households (8,983 individuals) in Vietnam were interviewed.

Purposive samples were selected for FGDs and in-depth interviews. Gender, health insurance membership, and location were taken into consideration when selecting the respondents to capture a wide range of experiences and views. In total, in China 26 FGDs were held with male and female NCMS members and non-members, 89 in-depth interviews with patients with catastrophic medical expenditure, health providers, health insurance policy makers and managers. In Vietnam, 26 FGDs were convened with members of different health insurance schemes and non-members. Sixteen in-depth interviews were conducted with health managers and health insurance managers.

### Data collection

Data collection was conducted from May to July 2006 by researchers from both countries. Standard structured questionnaires were developed in English for discussion and to ensure the maximum possible comparability between the two countries, and then translated into local languages for data collection. Questions related to this paper included: demographic information on individuals and households, rural health insurance membership, reported health service utilization (of outpatient service by those reporting illness in the last 4 weeks, and inpatient service in the last year). After receiving training, school teachers in Vietnam and postgraduate students in China acted as interviewers to visit the selected households and conduct the interviews. Completed questionnaires were carefully checked by quality supervisors immediately after the interview for quality assurance.

For qualitative study, semi-structured topic guides for FGDs and in-depth interviews were developed in a similar process as the questionnaire. Senior qualitative researchers from partner institutions acted as facilitators and interviewers. The interviews with health managers were conducted to explore rural health insurance policies, design and implementation processes. Selected rural residents were asked questions about factors affecting their health seeking behaviour, their perceptions and experiences of health insurance, and the reasons of choosing their health insurance membership.

### Data analysis

The quantitative data analysis focuses on a comparison of outpatient and inpatient service utilization between health insurance members and non-members in different income groups. In this study, outpatient service utilization rate is defined as the number of people who used outpatient services in the last 4 weeks as a percentage of total number of individual interviewees. Inpatient service utilization rate is defined as the percentage of total respondents who were hospitalized in the last 12 months. In calculating these two indicators, the numerators were number of patients, rather than number of service episodes, that is, even if a patient had more than one outpatient visits or hospitalization episodes, s/he was only counted once in the analysis. Reported household annual income per capita was used as a proxy for socio-economic status. Income groups were defined based on the reported household annual income per capita. From the lowest to the highest annual income per capita in each country, we divided the whole country sample into three equally sized groups: low income group, middle income group and high income group. Analysis was conducted using SPSS 14.0 and Stata 8.0.

All interviews were tape recorded with the permission of participants and were then transcribed and word processed and entered into MaxQDA. The 'framework' approach was used to analyze the qualitative data [15]. Researchers read through the transcriptions and listed the recurring viewpoints and the common themes from the data. This formed the basis of the thematic framework. Each segment of the text was categorised and coded using this framework. Segments relevant to each theme were then charted to identify majority and minority views, enabling interpretation and explanation. Findings relevant to views and experiences of health insurance and reasons for service utilization and non-utilization were compared among different sampled groups.

## Results

### Health insurance coverage

There was a large difference in health insurance coverage between the two countries. In China, the NCMS coverage rate in the six counties was relatively high, 85% in Ningxia and 91.3% in Shandong, while Vietnam had a lower coverage of around 50% including both voluntary and compulsory schemes (Table 1).

**Table 1:** Health insurance coverage in rural China and Vietnam

		Compulsory HI %(n/N)	Voluntary HI %(n/N)	Total %(n/N)
China	Shandong	N/A*	91.3% (10764/11789)	91.3% (10764/11789)

	Ningxia	N/A*	85.0% (9217/10847)	85.0% (9217/10847)

Vietnam	Hai Duong	25.7% (1098/4274)	23.7% (1012/4274)	49.4% (2110/4274)

	Bac Giang	27.2% (1280/4709)	25.5% (1202/4709)	52.7% (2482/4709)

Data source: Household survey, 2006

* The study areas were in rural China where there is no compulsory health insurance.

### Health needs between health insurance members and non-members

Health insurance members had slightly higher age (35.5) than non-members (30.8) in China while these two groups had similar age in Vietnam. Male and female respondents had a relatively equal share in the sample (Table 2).

**Table 2:** Health needs between members and non-members in China and Vietnam

	China		Vietnam	
	**Member**	**Non-members**	**Member**	**Non-members**

Age (mean and standard deviation)	35.5 (19.8)	30.8 (20.6)*	33.4 (21.9)	33.9 (20.1)

% of Male	49.5%	54.0%*	48.2%	50.2%

% of female	50.5%	46.0%	51.8%	49.8%

Prevalence of illness in the last 4 weeks	24.3%	26.2%	20.2%	19.1%

Prevalence of chronic disease in last 12 months	17.4%	17.4%	20.9%	17.1%

Data source: Household survey, 2006

* *P *< 0.05, significant difference between members and non-members

Table 2 also shows that there was no significant difference in prevalence of illness in the last 4 weeks and chronic disease in the last 12 months between health insurance members and non members in China. In Vietnam, the prevalence of chronic disease in the previous 12 months was slightly (but statistically significant) higher among the health insurance members (20.9%) than the non-members (17.1%), but no significant difference in the prevalence of illness in the last 4 weeks was identified between the two groups.

### Equity in utilization of health services

Table 3 presents utilisation of outpatient services between members/non-members and income groups in China and Vietnam. In China, there was no difference in utilisation of outpatient services between members and non-members regardless of their household income.

**Table 3:** Outpatient service utilization rate in the last 4 weeks by income groups and health insurance membership in six counties of China and 4 districts of Vietnam (2006)

	China			Vietnam		
**Income groups**	**Member %** **(n/N)**	**Non-Member %** **(n/N)**	**OR**	**Member %** **(n/N)**	**Non-Member %** **(n/N)**	**OR**

Low	10.7% (517/4825)	10.0% (67/670)	1.1	17.3% (164/950)	13.8% (112/810)	1.3*

Middle	9.1% (989/10843)	10.7% (82/763)	0.8	11.8% (261/2211)	11.2% (282/2515)	1.1

High	8.7% (455/5221)	9.5% (29/306)	0.9	10.6% (127/1202)	10.0% (129/1295)	1.1

Data source: Household survey, 2006. 8 individuals in China had miising values in reporting their sertive utilization, therefore the total sample size is 22628.

* *p *< 0.05, according to Chi Square test

Vietnam had a similar level of outpatient service utilization as in China. In the low income group, health insurance members used more outpatient services (17.3%) than non-members (13.8%), while in the middle and high income groups, there were no significant differences in outpatient service utilization between members and non-members.

Analysis of inpatient service utilisation showed very different patterns between the two countries (Table 4). In China, more NCMS members from the high income group used impatient services (4.8%) than non-members (1.3%, *P *< 0.05). In the low income group, however, though the impatient service utilization rate was slightly higher among NCMS members than that in non-members, the difference was not statistically significant (*p *= 0.078). No difference was identified in the middle income group.

**Table 4:** Inpatient service utilization rate in the last year by income groups and health insurance membership in six counties of China and 4 districts of Vietnam (2006)

	China			Vietnam		
**Income groups**	**Member % (n/N)**	**Non-Member % (n/N)**	**OR**	**Member % (n/N)**	**Non-Member % (n/N)**	**OR**

Low	5.2% (249/4825)	3.6% (24/670)	1.5	9.9% (94/950)	4.2% (34/810)	2.5*

Middle	4.6% (503/10843)	4.6% (35/763)	1.0	7.2% (158/2211)	4.5% (113/2515)	1.6*

High	4.8% (253/5221)	1.3% (4/306)	3.8*	6.2% (75/1202)	4.6% (60/1295)	1.4*

Data source: Household survey, 2006

* *p *< 0.05, according to Chi Square test

In Vietnam, the overall inpatient service utilization rate (5.9%) was higher than that in China (4.7%). In all three income groups in Vietnam, health insurance members were significantly more likely to utilise inpatient services. The low income group had the highest odds ratio (2.5) than the middle and high income groups (1.6 and 1.4 respectively).

### Experiences and problems of accessing and utilising health services: qualitative findings

#### Limited financial protection and complicated reimbursement procedure in China

The most frequently mentioned reason of not using health services was the high medical cost and, for members, the limited financial protection provided by NCMS. Although NCMS policy states that it will cover up to 50% of the medical expenses, members reported that the actual reimbursement level was much lower. Most health managers perceived it as impossible to significantly relieve economic burdens for NCMS members, given the low reimbursement rate and low ceilings applied to expenditure. Members who saw themselves as poor expressed the view that the low level of reimbursement they could receive would not help their economic situation. They also had to pre-pay all the medical expenses before they could get a small amount of money reimbursed. As a result, they would prefer not to utilise health services at all if possible.

In addition, the complicated reimbursement process acted as another main barrier to NCMS members using their health insurance to reduce their healthcare costs. To obtain reimbursement, they explained that they needed to show the NCMS enrolment certificate, receipt for all medical expenses, proof of residence status (*hukou*), and sometimes a referral document. Most members were not clear what they were supposed to take to the NCMS office for reimbursement. Participants also complained that they usually needed to wait a long time or make many journeys to get reimbursement, and sometimes the money reimbursed did not even cover the cost of this travel (see quotes below).

*"A patient who has an illness spends 20000-30000 Yuan, and will be reimbursed 2000 - 3000 Yuan. This amount of money cannot solve his trouble. If a patient spends 2000-3000 Yuan, he/she only can get reimbursement of 200-300 Yuan. As the procedure for reimbursement is complicated and the reimbursement proportion is low, some patients are not willing to go through the process." *(from Interview with County health manager, Shandong)

*"I heard from a neighbour that he visited the reimbursement office 3-4 times, but just got back 70 Yuan from an expenditure of 5000." *(from FGD NCMS member, Ningxia)

#### Barriers to receiving and using health insurance card in Vietnam

Members of VHI schemes complained of a number of barriers to both receiving and using their health insurance cards. Most VHI members reported that the waiting period between paying money and receiving health insurance cards was very long; it took 2 to 3 months in some areas. During this time, these people were unable to use the health insurance scheme. Health insurance managers explained this was because of a delay in collecting money from the communes and the administration process at province level. It was also reported that information on the health insurance cards was often incorrect, leading to further delays before members could use their cards.

Even after receiving health insurance cards, people may decide not to use the cards when using health services. Many members reported they would use health insurance cards for inpatient care, but not for outpatient care. Both CHI and VHI members perceived that health insurance members received poorer quality of services than non-members did. Some complained they were prescribed only limited types and amounts of medicine; others recounted that they had to wait longer than those without health insurance. Health insurance members reported that these barriers negatively affected their use of formal health services. Going to private drug sellers were the most common pattern of health care service utilization among the insured participants (see quotes below).

*"People always complain that they have given money to the health insurance agency, but still do not receive their health insurance cards even after 2 months" *(from interview with a representative of Commune Women's Association)

*"We have to wait for the list from every commune. Then we combine the list, print the cards and deliver at the same time. In communes where persons in charge are not enthusiastic or where people have low awareness on health insurance, it takes a longer time to disseminate information and convince people to buy voluntary health insurance." *(from interview with a member of Vietnam Social Security in province)

*"We buy health insurance just in case we need to be in hospital. For outpatient care, it will be quicker if we pay directly. " *(from FGD with voluntary health insurance member)

*"I came to commune health station for consultation. I have a bad cough and need to use antibiotics. But the doctor only gave me some "normal" medicine and some antibiotics tablets that I can take for 3 days only, and then I have to pay for another amount of antibiotics. " *(from FGD with member of the compulsory health insurance for the poor).

*"It is quite different between insured and paying patients. For paying patients, physicians serve [them] immediately. Therefore I buy medicine for self- treatment at home. " *(from FGD with voluntary health insurance members).

*"My son had a cough, I brought him to see the doctor in the hospital, but then he did not see my son. He treated the paying patients first. My son was very sick but we had to wait from early in the morning until noon time. " *(from FGD with parents/carers of school children with voluntary health insurance membership)

## Discussion

### Limitations of the study methodology

First, rural health insurance in Vietnam includes different fragmented schemes including CHI, VHI, the health care fund for the poor and others, but due to sample size limitations, this study combined them together and did not analyze their utilization separately. Second, the high coverage rate of NCMS in rural China resulted in a large difference in the sample size of NCMS members and non-members, which created difficulties in comparing utilisation between members and non-members in China. This imbalance in the sample size also means that it is not possible to analyze the data by provinces, districts or counties, especially for inpatient service utilization. In addition, due to the observational nature of study design, the association between health insurance and service utilization may not be causal relationship.

### Adverse selection in health insurance

NCMS in China is officially a voluntary scheme. Vietnam also has a component of VHI. This may raise concerns about adverse selection, whereby people with higher health needs are more likely to join the health insurance than those with lower health needs. Adverse selection introduces bias in comparing the health service utilization between health insurance members and non-members. Considering this, we have compared the demographic background and the prevalence of disease between health insurance members and non-members. The results showed no significant difference in China, which is possibly due to the very high coverage rate of NCMS. In Vietnam there was slight but statistically significant difference in chronic disease prevalence but no difference in acute illness prevalence between health insurance members and non-members. This suggests that there is an element of adverse selection of health insurance members in Vietnam.

### Different routes towards achieving universal healthcare coverage

China and Vietnam have chosen different routes towards achieving the goal of universal healthcare coverage by developing their health insurance systems. China has prioritised general population coverage in the development of NCMS and has been very successful in this regard. Most counties started NCMS from scratch in 2004 or 2005 and had already achieved a high coverage of 85% to 90% in 2006. These levels may be slightly higher than average: another recent study of 10 counties found an average coverage level of 82.7% in 2005 [16], whilst Ministry of Health statistics put the national enrolment level at 85.7% in 2007 [17]. A high level of political commitment has been an important factor enabling this remarkable achievement [17,18]. Although NCMS is in theory a voluntary scheme, significant efforts have been made to encourage the rural residents to join the scheme, which in some cases amounted to coercive measures, in order to reach coverage targets. Subsidies from national and local governments have enabled a relatively low individual premium level, which contributes to the high coverage rate [18]. Vietnam, following a more common social health insurance model, started developing health insurance to cover certain population groups, such as civil servants, the poor, and school pupils. International experiences from industrialised countries suggests that this model of social health insurance tends to be relatively slow in reaching the goal of universal coverage, with the shortest timeframe to date at 26 years in South Korea and the longest at more than 100 years in Germany [1].

However, high population coverage doesn't automatically mean equal access to and utilization of health services. Some of the insured may not use the health service because of unaffordable high co-insurance payments and thus low financial protection, as is the case in China, or because of dissatisfaction with quality of services provided in assigned facilities, particularly for health insurance members, as in Vietnam. Universal coverage means not only the breadth of coverage - the proportion of the population that enjoys social health protection, but also the depth of coverage - the range of benefit package, and the height of coverage - portion of healthcare costs covered [19].

### Health insurance policy and equity in health care utilization

This study found that rural health insurance members were more likely to utilise inpatient services but membership had very limited impact on outpatient care utilization, especially in China. The limited impact on outpatient service utilisation is likely to be due, at least in part, to the benefit package designs [20]. The central stated goal of developing the rural health insurance scheme in China is to provide financial protection to individuals and households from catastrophic expenditures due to major illness [21]. Therefore the benefit package in the early years of NCMS mainly focused on inpatient services with a relatively higher reimbursement rate. Since the costs of outpatient care are seen as less likely to reach catastrophic level, outpatient services are hardly covered by NCMS in most counties. Outpatients either had no reimbursement at all, or had a much lower reimbursement rate than that of inpatient services. However, as the NCMS policy is quickly moving on with increasing government subsidies, most counties in China are trying to expand NCMS benefit package to cover outpatient services. In Vietnam, although both inpatient and outpatient service at public facilities were covered in the health insurance policy, our qualitative data showed that people tended not to use their health insurance card for outpatient service due in part to the limited benefit package, for example limited choice of medicines and delayed treatment for health insurance members. Another study found that outpatient care provided to the insured in Vietnam were of inferior quality [4].

This study also found that the low income group benefited more from health insurance in terms of using inpatient services than the high income group in Vietnam, but less in China. This finding in China is supported by a recent study of 10 counties [16]. There are two likely explanations for this. First, high co-insurance payments and the consequent financial burden will make a greater contribution towards decision not to use services for the poor, for whom even small payments for health care can have catastrophic consequences [14]. The escalating medical costs and high financial burdens placed on individuals and households due to out of pocket payments for healthcare in China have long been discussed among academics and recognized by policy makers [22-24]. The study found that the reimbursement level offered by NCMS was perceived as too low by all stakeholders. We have reported quantitative findings on the actual reimbursement level in the study counties, which support these views, in other papers [25,26]. Another study of 10 counties found that the average co-insurance rate for all types of care ranged from 60% to 66% depending on the level of services used, and also showed that the average cost of health care per visit had increased as a result of NCMS [16]. Poor people are less likely than better off people to be able afford large co-payments in the context of high medical costs, especially where they have to pay the whole cost before they can get a small amount of money reimbursed. They are therefore less likely to use services at times of need. In Vietnam, although the same concern of escalating medical expenses has been raised and debated in the recent years [4,27], the situation is not as serious as in China. The co-payment level in Vietnam was much lower than in China. This may further reduce the financial burden faced by the poor people. The Health Care Fund for the Poor has been found to increase utilization of health services, especially inpatient services [28].

Second, both China and Vietnam implemented pro-poor policies in their rural health insurance schemes, but using different approaches. The Chinese central government subsidized the less developed western and central provinces. All people living in the province, regardless of their socio-economic status, pay the same premium, and enjoy the same benefit package. The increasing disparity in income and ability to pay between households within provinces did not receive sufficient attention when designing the NCMS policy. However, in addition to NCMS, Chinese government also implemented a Medical Financial Assistance (MFA) scheme to support the poor households to afford their catastrophic medical expenditures [29]. In Vietnam, however, the central government subsidies were targeted at individuals living below the poverty line, who were identified and authorized by local government. This study suggests that the approach adopted in Vietnam has a greater impact on equity in health service utilisation between the poor and non-poor within the study localities, although some caution is needed with regard to the slight element of adverse selection. Further comparative monitoring of equity in access to health services during the rapid development in health insurance policy in both countries is needed to inform mutual learning.

## Competing interests

The authors declare that they have no competing interests.

## Authors' contributions

All authors contributed to the design of the study. XL, BY, NKP, FY and DDT collected and analyzed the research data. XL and RT drafted the manuscript. ST made important contributions to the revision of the paper. All authors read and approved the final manuscript.
